# Supplementary material for: An SH3-binding allosteric modulator stabilizes the global conformation of the AML-associated Src-family kinase, Hck
Source: J Biol Chem. 2024 Dec 13;301(1):108088. doi: 10.1016/j.jbc.2024.108088 (PMC11786751; doi:10.1016/j.jbc.2024.108088)
Supplement: Supplemental Figs. S1–S7 [file mmc2.pdf]

Supporting Information

An SH3-binding Allosteric Modulator Stabilizes the Global Conformation of the AML-associated Src-Family Kinase, Hck

Ari M. Selzer, Gabriella Gerlach, Giancarlo Gonzalez-Areizaga, Thomas E. Wales, Prema Iyer, John R. Engen, Carlos Camacho, Rieko Ishima, and Thomas E. Smithgall

Contents:

Synthesis and analytical characterization of PDA1 and PDA2

Figure S1: Complete HSQC NMR spectra of the Hck SH3-SH2-Linker protein in the presence of PDA1 and PDA2

Figure S2: Assignment of the indole N-H resonance of Hck SH3-SH2 Trp118

Figure S3: Linker effect on SH3-SH2 chemical shifts, assignment of linker W254 indole resonance, and DMSO control

Figure S4: HSQC NMR spectra of the Hck SH3 domain in the presence of PDA1 and PDA2

Figure S5: PDA1 and PDA2 NMR titration analysis with the Hck SH3 domain

Figure S6A: HDX-MS difference maps for near-full-length Hck in the presence of PDA1 and PDA2 (SH3, SH2, linker)

Figure S6B: HDX-MS difference maps for near-full-length Hck in the presence of PDA1 and PDA2 (kinase domain)

Figure S7: Effects of A-419259 and PDA2 on the viability of TF-1 cells expressing wild-type and active Hck

Synthesis and analytical characterization of PDA1 and PDA2.

Synthesis of pyrimidine diamine 1 (PDA1).

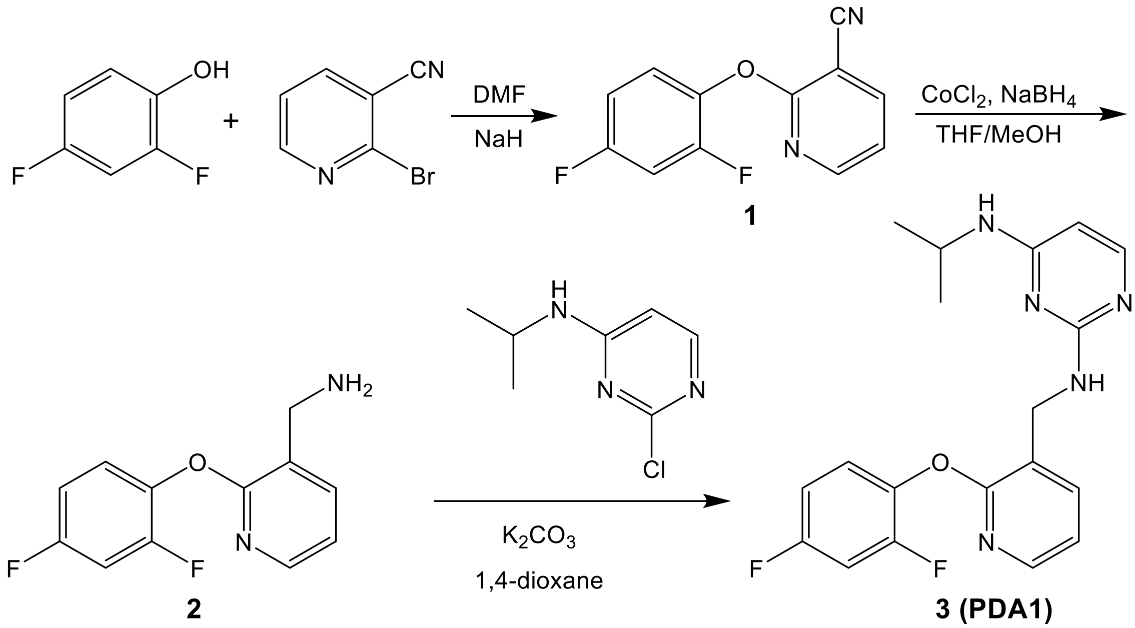

2-(2,4-difluorophenoxy)nicotinonitrile, 1. The phenol (0.746 g, 5.74 mmol) was dissolved in anhydrous DMF (30 mL) under Ar. NaH (60%, 0.262 g, 6.56 mmol) was added portion wise and and the mixture was stirred at room temperature for 1 h. The nitrile (1.000 g, 5.46 mmol) was added, the reaction stirred at room temperature for 2 h, and partitioned in EtOAc and water. The aqueous layer was extracted thoroughly with EtOAc. The organic layer was washed with water, brine, dried over Na_2SO_4 and solvents were evaporated to give a brown viscous residue. The residue was then purified by column chromatography on silica (ISCO-Rf, 0-50% EtOAc/hexanes) to give **1** as a white solid (0.719 g, 57%). ^{1}H NMR (400 MHz, $CDCl_3$): δ 6.93-7.00 (m, 2H), 7.14 (dd, 1H, J = 4.8 Hz, J = 7.2 Hz), 7.22-7.27 (m, 1H), 8.03 (dd, 1H, J = 2.0 Hz, J = 7.6 Hz), 8.29 (dd, 1H, J = 2.0 Hz, J = 5.2 Hz).

(2-(2,4-difluorophenoxy)pyridin-3-yl)methanamine, 2. A solution of nitrile **1** (0.250 g, 1.077 mmol) in THF/MeOH (1:1, 25 mL) was stirred at 0°C and $CoCl_2$ (0.419 g, 3.230 mmol) portion wise followed by $NaBH_4$ (0.407 g, 10.767 mmol). The mixture was stirred at 0°C for 20 min, filtered through Celite, and the Celite was washed thoroughly with dichloromethane. The organic layer was then treated with water and the water-organic mixture was filtered through Celite. The organic layer was separated, dried over Na_2SO_4 and the solvent evaporated to give a brown viscous residue. The residue was purified by silica chromatography (ISCO-Rf, 0-15% MeOH/dichloromethane) to yield **2** as a brown gel (0.150 g, 59%). LC-MS (ESI) m/z: calculated 236.22; observed 237.2 (M+1).

***N*2-((2-(2,4-difluorophenoxy)pyridin-3-yl)methyl)-*N*4-isopropylpyrimidine-2,4-diamine, 3 (PDA1).** The amine **2** (0.150 g, 0.635 mmol), anhydrous K_2CO_3 (0.263 g, 1.905 mmol), and 2-chloro-N-isopropyl-4-pyrimidinamine (0.163 g, 0.952 mmol) were combined with 1,4-dioxane (5 mL) in a sealed tube and heated to 135 °C for 16. The dioxane was evaporated and the residue purified twice by silica chromatography (ISCO-Rf, 0-15% MeOH/dichloromethane) to give **3** as a tan solid (0.051 g, 28%). ^{1}H NMR (600 MHz, DMSO): δ 1.04 (s, 6H), 3.97 (s broad, 1H), 4.54 (d, 2H, J = 6.0 Hz), 5.70 (s, 1H), 6.75 (s broad, 1H), 6.95 (s broad, 1H), 7.07-7.15 (m, 2H), 7.37-7.44 (m, 2H), 7.62-7.66 (m, 2H), 7.90 (d, 1H, J = 3.0 Hz). ^{13}C NMR (150 MHz, DMSO): δ 22.34, 104.91, 105.06, 105.09, 105.24, 111.52, 111.54, 111.67, 111.69, 119.10, 123.34, 125.20, 125.26, 137.10, 137.19, 144.28, 153.36, 153.45, 155.01, 157.96, 158.03, 159.41, 159.57, 159.65, 161.86, 162.03.
^{19}F NMR (500 MHz, DMSO): δ -123.73, -114.35.

Synthesis of pyrimidine diamine 2 (PDA2).

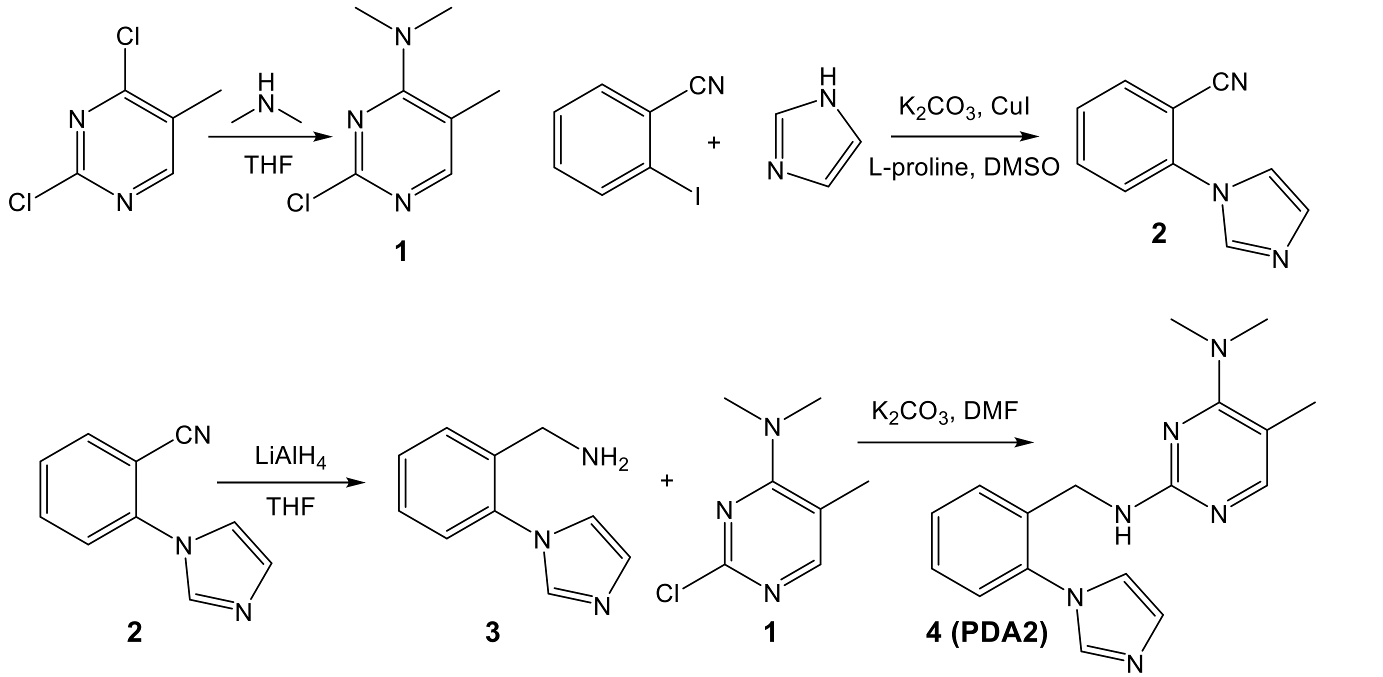

2-chloro-N,N,5-trimethylpyrimidin-4-amine, 1. 2,4-dichloro-5-methylpyrimidine (1.000 g, 6.135 mmol) was dissolved in THF (16 mL) under Ar. The reaction vessel was then cooled to 10 °C and dimethylamine (2M in MeOH, 6.6 mL, 13.3 mmol) was added and reaction was left to stir for 2.5 hours. Solvents were evaporated to give a white solid which was purified by silica chromatography (ISCO-Rf, 0-50% EtOAc/hexanes); 0.893 g, 85%. ^{1}H NMR (600 MHz, $CDCl_3$): δ 2.27 (s, 3H), 3.14 (s, 6H), 7.83 (s, 1H).

2-(1H-imidazol-1-yl)benzonitrile, 2. 2-iodobenzonitrile (1.000 g, 4.366 mmol), imidazole (0.357 g, 5.240 mmol), K_2CO_3 (1.208 g, 8.733 mmol), CuI (0.083 g, 0.437 mmol), and L-proline (0.101 g, 0.873 mmol) were combined in DMSO (10 mL) in a sealed vial and left to stir at 120 °C for 23 hours. The reaction was then partitioned in EtOAc and water and NH_4OH was added to break the emulsion. The aqueous layer was extracted with EtOAc. The organic layer was washed with water and brine, and dried over Na_2SO_4 and solvents were evaporated to give a light yellow solid. The solid was then purified by silica chromatography (ISCO-Rf, 0-100% EtOAc/dichloromethane) to give the product as a white solid (0.358 g, 48%). ^{1}H NMR (600 MHz, $CDCl_3$): δ 7.28 (s, 1H), 7.37 (s, 1H), 7.47 (d, 1H, J = 7.8 Hz), 7.54 (t, 1H, J = 7.8 Hz), 7.75 (t, 1H, J = 7.8 Hz), 7.84 (d, 1H, J = 7.8 Hz), 7.87 (s, 1H).

(2-(1H-imidazol-1-yl)phenyl)methanamine, 3. A solution of nitrile **2** (0.150 g, 0.887 mmol) in THF (6 mL) under Ar was combined with $LiAlH_4$ (2M in THF, 0.887 mL, 1.773 mmol) dropwise at 0 °C and the reaction mixture was stirred and allowed to come to room temperature over 1.5 h. The reaction was re-cooled back to 0 °C and quenched with NH_4Cl dropwise until effervescence stopped. Na_2SO_4 was added and the reaction was left to stir at room temperature for 30 minutes. The reaction was then filtered through Celite, washed thoroughly with EtOAc/dichloromethane (1:1) and solvents evaporated to give an amber liquid. The liquid was purified by silica chromatography (ISCO-Rf, 0-15% MeOH/ dichloromethane) to give the product as a yellow residue (0.121 g, 79%). LC-MS (ESI) m/z: calculated (173.22); observed (M+1=174.2).

N2-(2-(1H-imidazol-1-yl)benzyl)-N4,N4,5-trimethylpyrimidine-2,4-diamine, 4 (PDA2). Amine **3** (0.121 g, 0.699 mmol), anhydrous K_2CO_3 (0.290 g, 2.096 mmol), and pyrimidine **1** (0.180 g, 1.048 mmol) were combined in DMF in a sealed vial and stirred at 140 °C for 15 h. DMF was evaporated, the reaction partitioned with water and MeOH/dichloromethane (1:10) and the aqueous layer extracted with EtOAc. The organic layer was washed with brine, dried over Na_2SO_4, and solvents evaporated to give a yellow liquid. The liquid was purified by silica chromatography (ISCO-Rf, 0-100% EtOAc/dichloromethane, 0-15% MeOH/dichloromethane) to yield 74 mg of a yellow viscous residue. The residue was repurified by silica chromatography (ISCO-Rf, 0-15% MeOH/dichloromethane containing 0.1% Et_3N) to give a tan solid (0.018 g, 8%). ^{1}H NMR (600 MHz, DMSO): δ 2.06 (s, 3H), 2.86 (s, 6H), 4.26 (d, 2H, J = 6.0 Hz), 6.92 (s broad, 1H), 7.10 (s, 1H), 7.29 (d, 1H, J = 7.8 Hz), 7.35 (t, 1H, J = 7.2 Hz), 7.41 (t, 1H, J = 7.2 Hz), 7.44 (s, 1H), 7.49 (d, 1H, J = 7.2 Hz), 7.57 (s, 1H), 7.85 (s, 1H). ^{13}C NMR (150 MHz, DMSO): δ 7.14, 17.17, 51.94, 121.19, 126.08, 127.26, 128.18, 128.37, 128.72, 135.40, 136.45, 137.85, 158.12, 160.07, 164.11. LC-MS (ESI) m/z: calculated (308.39); observed (M+1=309.4).

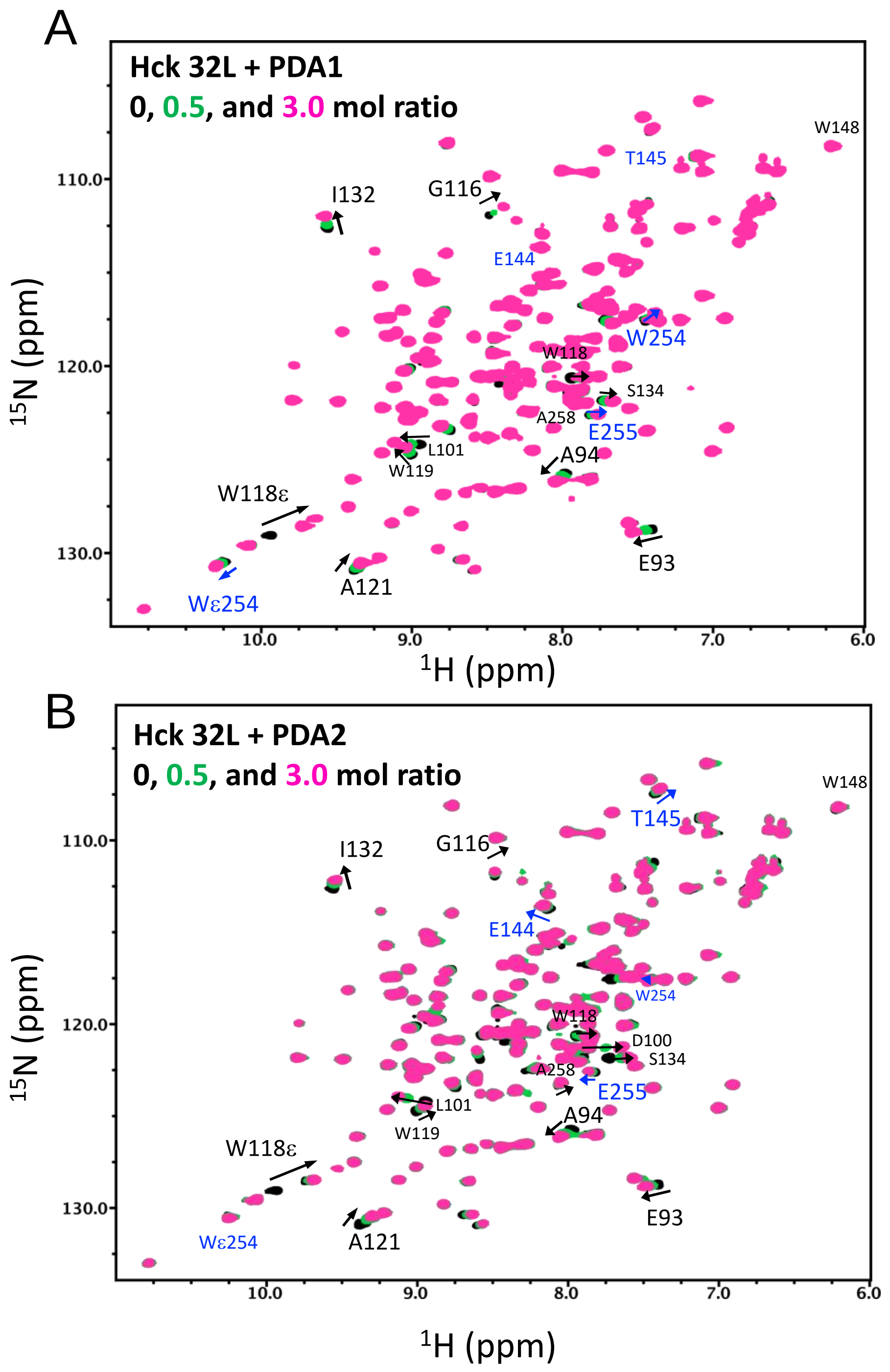

Figure S1: Complete HSQC NMR spectral changes of the Hck SH3-SH2-Linker protein upon PDA1 and PDA2 titration. An ^{15}N-labeled Hck SH3-SH2-linker protein (60 μM) was titrated with (A) PDA1 or (B) PDA2, at protein:ligand ratios of 1:0 (black), 1:0.5 (green) and 1:3 (pink) and ^{1}H-^{15}N HSQC spectra were recorded. In both panels, chemical shift perturbations (CSPs) were observed in a subset of ^{1}H-^{15}N resonances (highlighted by arrows), indicating the specific interaction of the compounds with the protein. Most of the N-H amide resonances that exhibit significant CSPs localize to the SH3 domain (black arrows). PDA1 induced a CSP of the Trp254 indole resonance in the linker region but did not influence residues in the connecter region between SH3 and SH2. PDA2, on the other hand, induced CSPs of connector amides including Glu144 and Thr145, but not Trp254 in the linker region (blue arrows). These results suggest that the shared pyrimidine diamine core in PDA1 and PDA2 interacts mainly with the SH3 domain while unique moieties in each compound affect the interdomain regions.

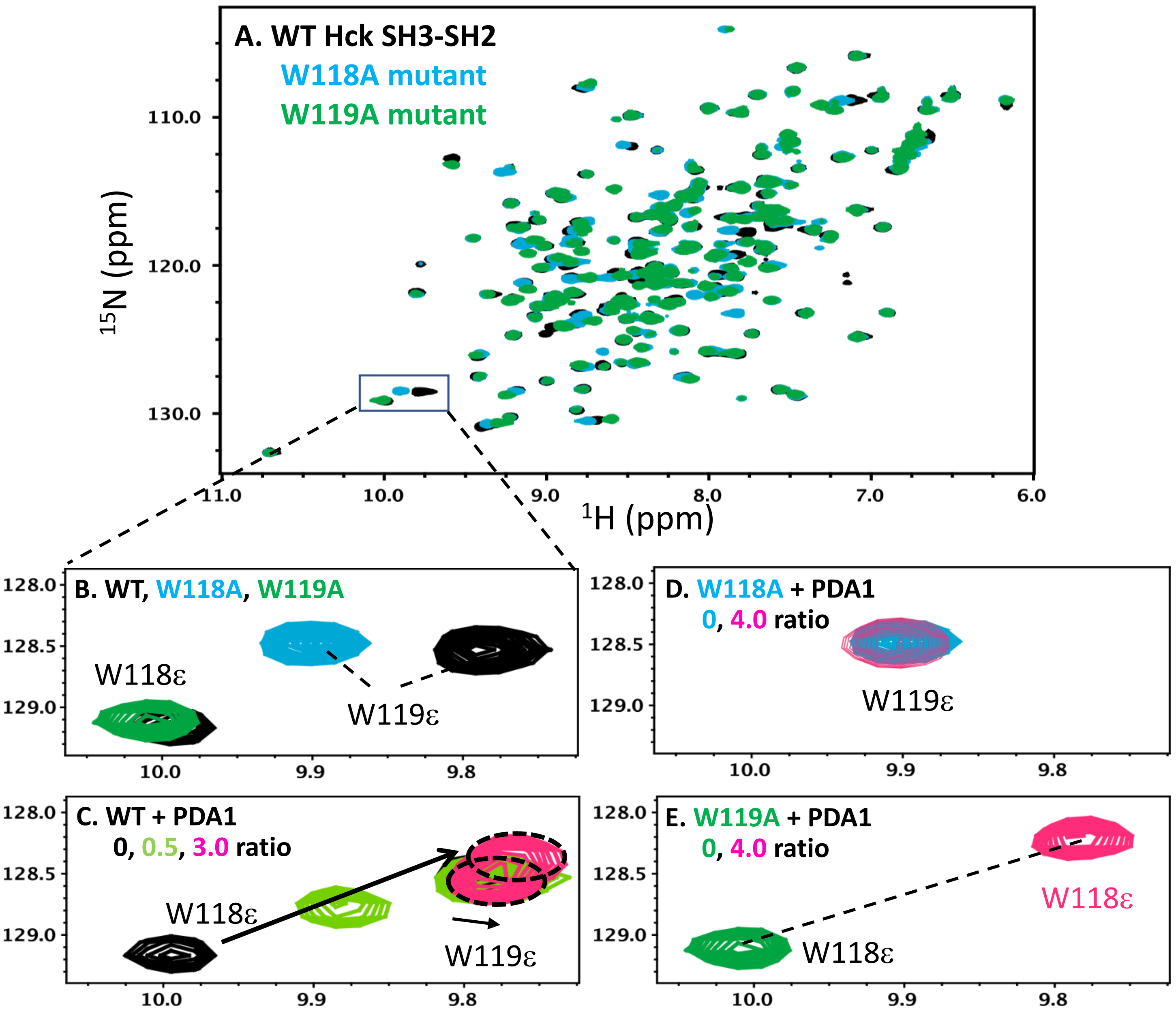

Figure S2: Assignment of indole N-H resonances of Hck SH3-SH2 Trp118 and Trp 119. A) Overlay of ^{1}H-^{15}N HSQC spectra of wild-type Hck SH3-SH2 (WT; black), the W118A mutant (cyan) and the W119A mutant (green), at Hck protein concentrations of 60 µM. B) Enlarged view of the tryptophan N-H indole (ε) region. C) Confirmation that the W118ε, and not the W119ε, resonance is significantly shifted upon PDA1 titration. D) Hck SH3-SH2 W118A mutant titration with PDA1, demonstrating no change of the shift of adjacent residue, W119. E) Titration of the SH3-SH2 W119A mutant with PDA1 exhibits a significant chemical shift in W118ε like wild type. Note that PDA2 also exhibited similar changes to W118ε (not shown). These observations assign the W118ε and W119ε chemical shifts in Hck SH3-SH2 and confirm that the SH3 W118 indole ring is critical for both PDA1 and PDA2 interaction. Note that the assignment of tryptophan indole resonances is consistent with that of a homologous Fyn SH3-SH2 protein.[1]

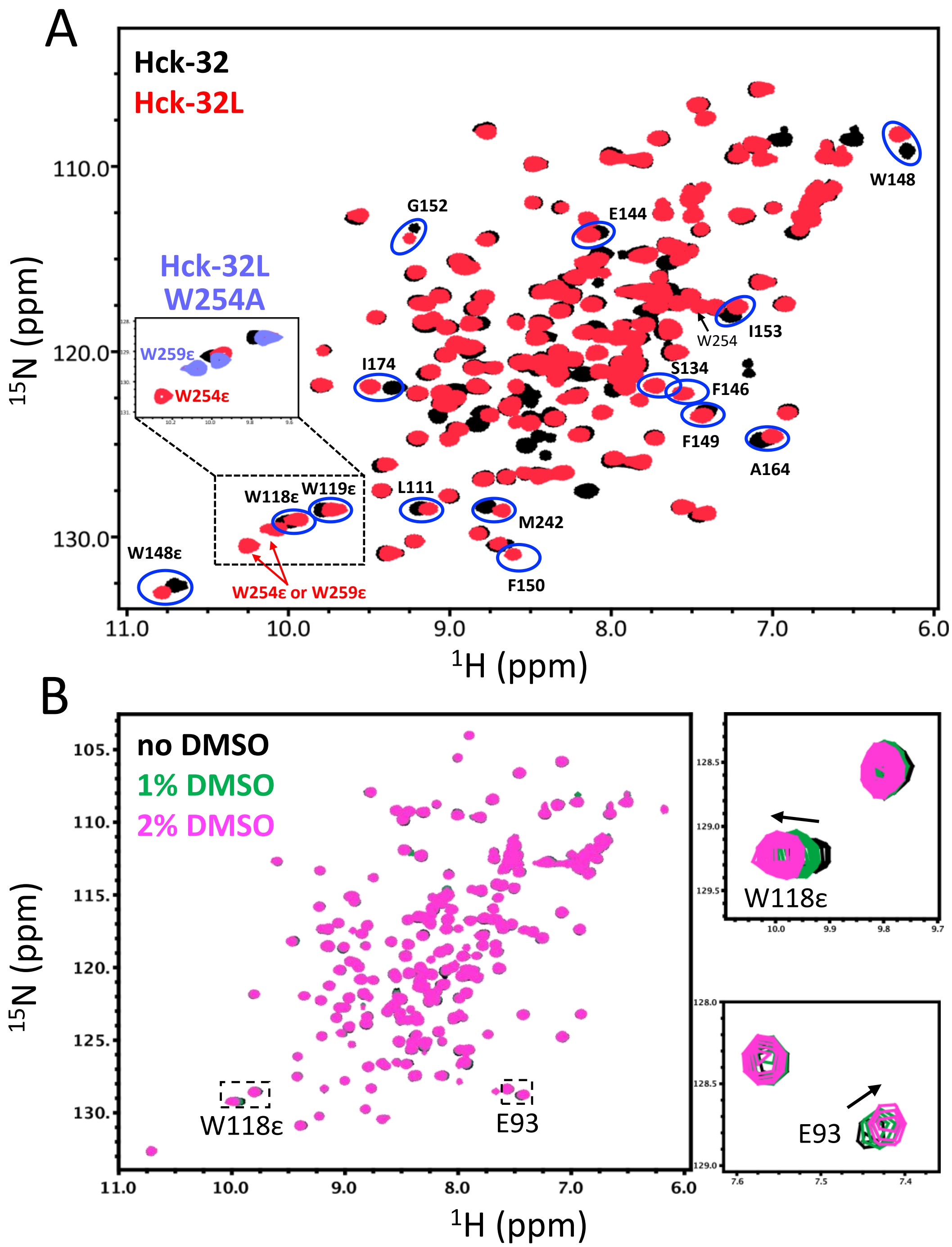

Figure S3: Linker effect on SH3-SH2 chemical shifts, assignment of linker W254 indole resonance, and DMSO control. A) Labeled residues exhibited significant chemical shift differences between the Hck SH3-SH2 (Hck-32; black) and SH3-SH2-linker (Hck-32L; red). The assignment of the backbone amides of Hck-32L is based on those of Jung *et al.*[2] Two tryptophan residues are present in the linker region, W254 and W259. The tryptophan indole N-H resonances (ε) were assigned by comparing the NMR spectra of the wild-type and W254A mutant forms of the Hck-32L protein (**light blue** resonances in the box). Several residues in the connector between SH3 and SH2 exhibit different chemical shifts between Hck-32 and Hck-32L (circled in blue), suggesting that the linker interacts with the SH3-SH2 region in solution. B) DMSO effect on NMR data was assessed using Hck-32. The overall ^{1}H-^{15}N HSQC spectral features of Hck-32 were not altered even at 2% DMSO. A few minor chemical shift perturbations (CSPs) were observed for some residues (e.g., W118 indole N-H and backbone amide of Glu93, right panels.) However, the directions of these small CSPs were opposed to those of observed with the PDA1 and PDA2 titrations, and thus do not affect the PDA titration results. Protein concentrations of Hck-32, Hck-32L, and Hck-32L-W254 were 60 µM, 60 µM, and 70 µM, respectively.

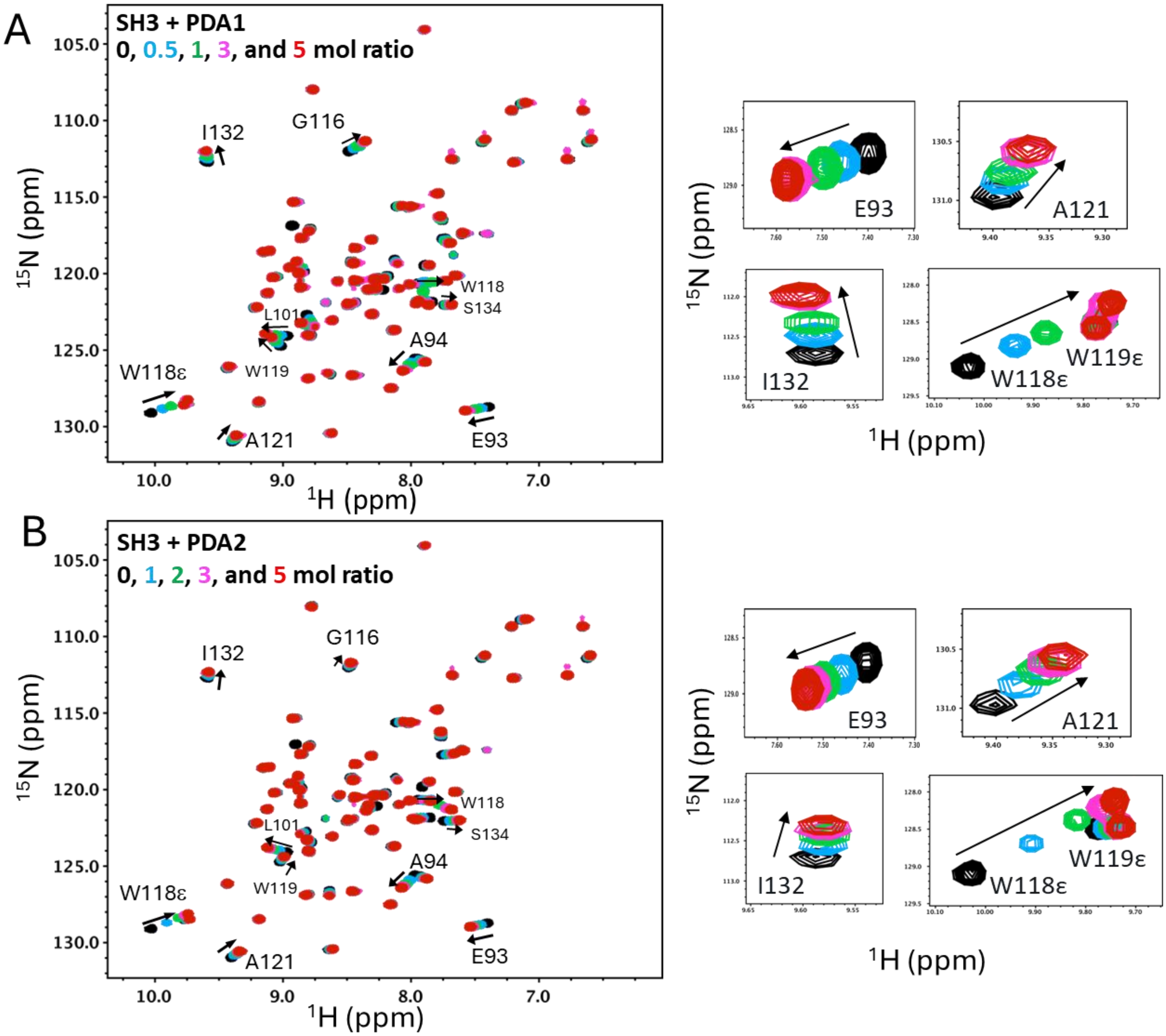

Figure S4: ^{1}H-^{15}N HSQC NMR spectral changes of the Hck SH3 protein upon PDA1 and PDA2 titration. An ^{15}N-labeled Hck SH3 protein (70 µM) was titrated with (A) PDA1 and (B) PDA2 at the molar ratios indicated. In both cases, chemical shift perturbations (CSPs) were observed in a subset of resonances (highlighted by black arrows), demonstrating a specific interaction of the compound with SH3 protein. The most substantial CSPs are enlarged on the right. These CSPs were essentially the same as those observed in the SH3 domain following PDA titration in the SH3-SH2-linker protein.

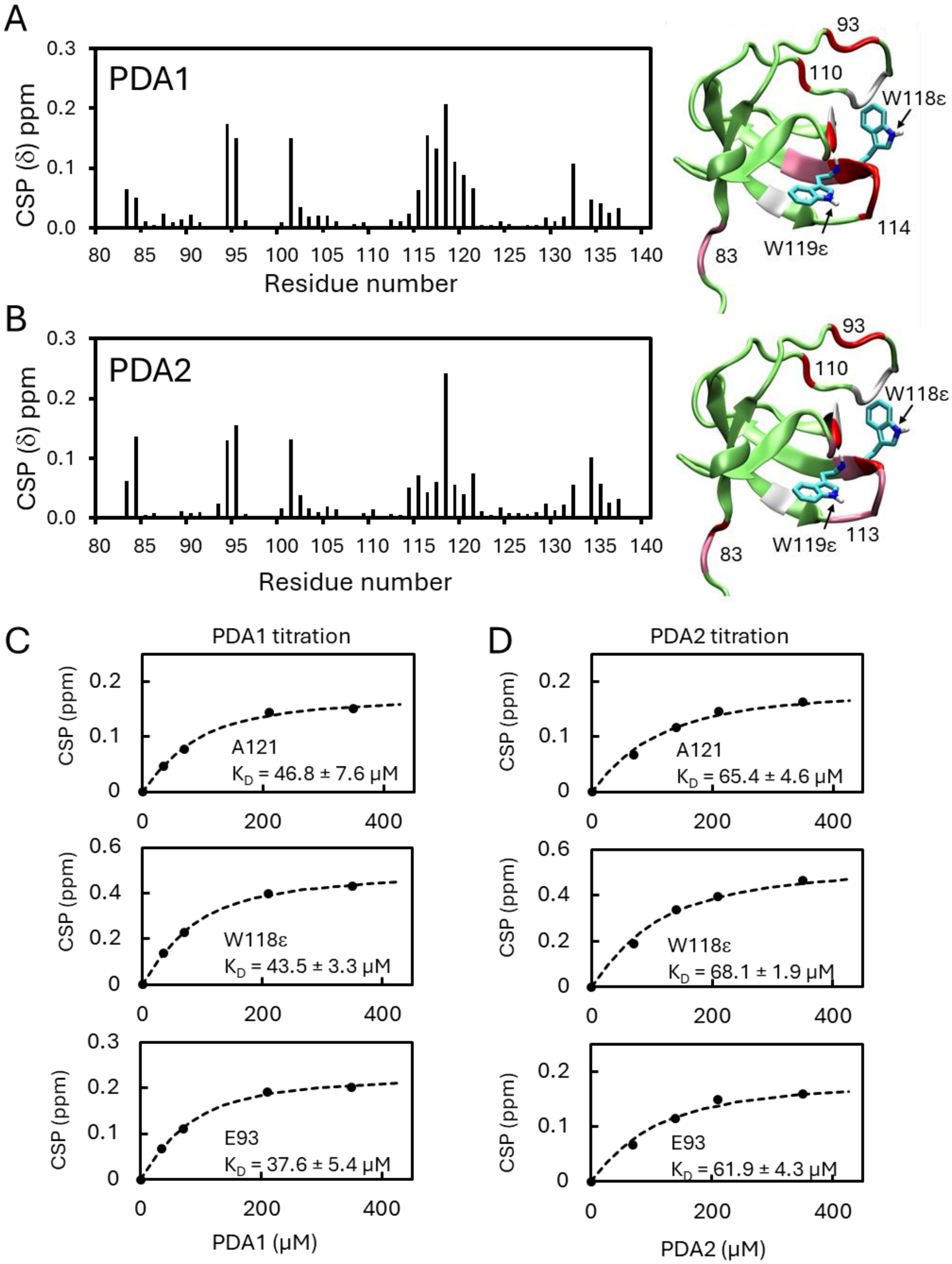

Figure S5: PDA1 and PDA2 NMR titration analysis with the Hck SH3 domain. Plots of CSPs at 1:3 SH3 protein:PDA ratio for A) PDA1 and B) PDA2 (SH3 concentration, 70 µM). CSPs are mapped on a ribbon structure of the Hck SH3 domain (*right*). Red, CSPs larger than one standard deviation above the average; pink, CSPs above the average; green, CSPs lower than the average; white, unassigned residues. CSPs were calculated using a combined shift of ^{1}H and ^{15}N with a weighting factor of 0.14 as per Williamson.[3] Titration curves for three selected residues (Glu93 and Ala121 backbone amides; Trp118 indole amide) with C) PDA1 and D) PDA2. All three resonances show saturation with average dissociation constants of 42.6 ± 4.7 µM (PDA1) and 65.1 ± 3.1 µM (PDA2).

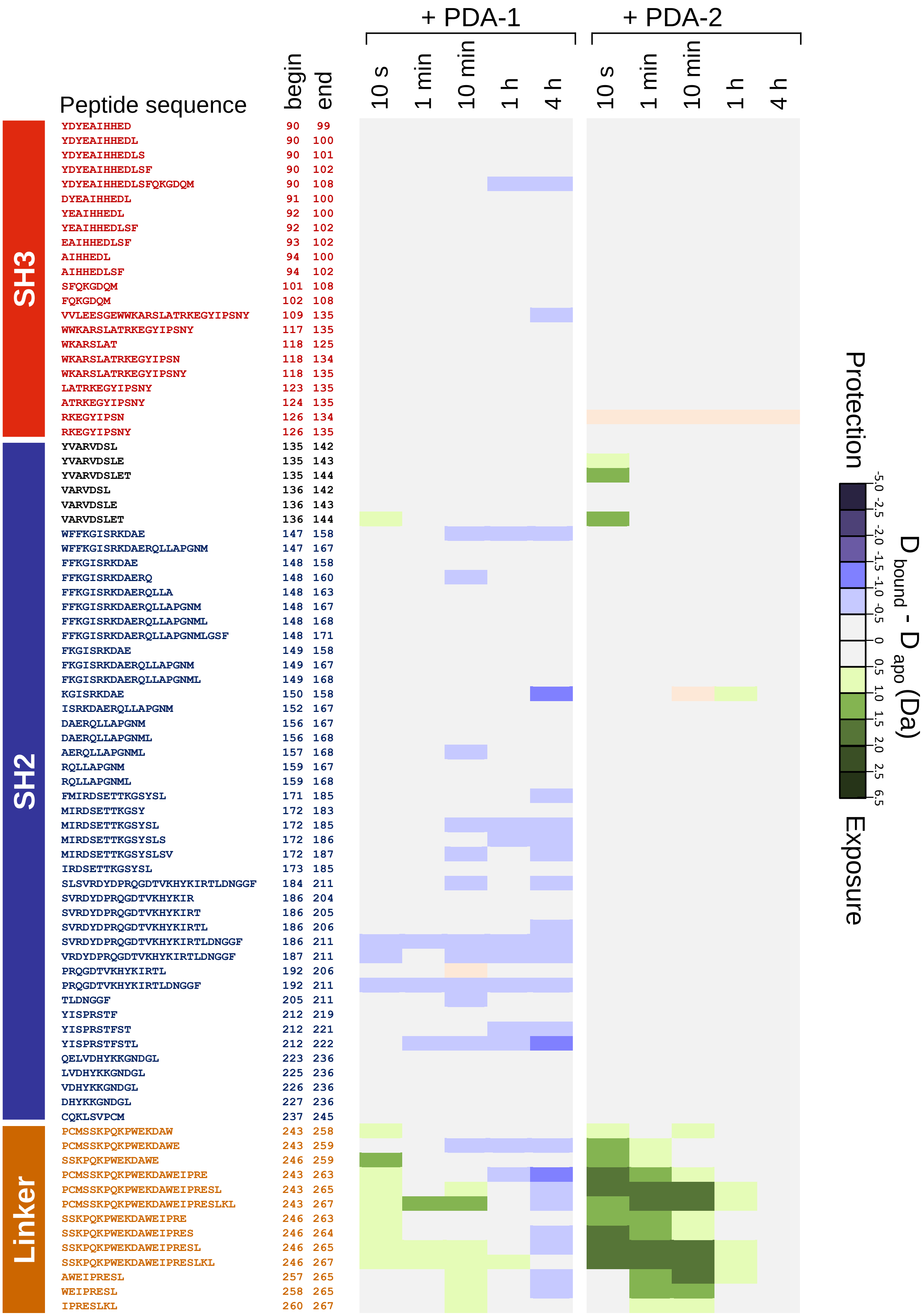

Figure S6A: HDX-MS difference maps for near-full-length Hck in the presence of PDA1 and PDA2. Data are shown for the SH3 and SH2 domains plus the SH2-kinase linker. Kinase domain peptides are shown in Figure S6B.

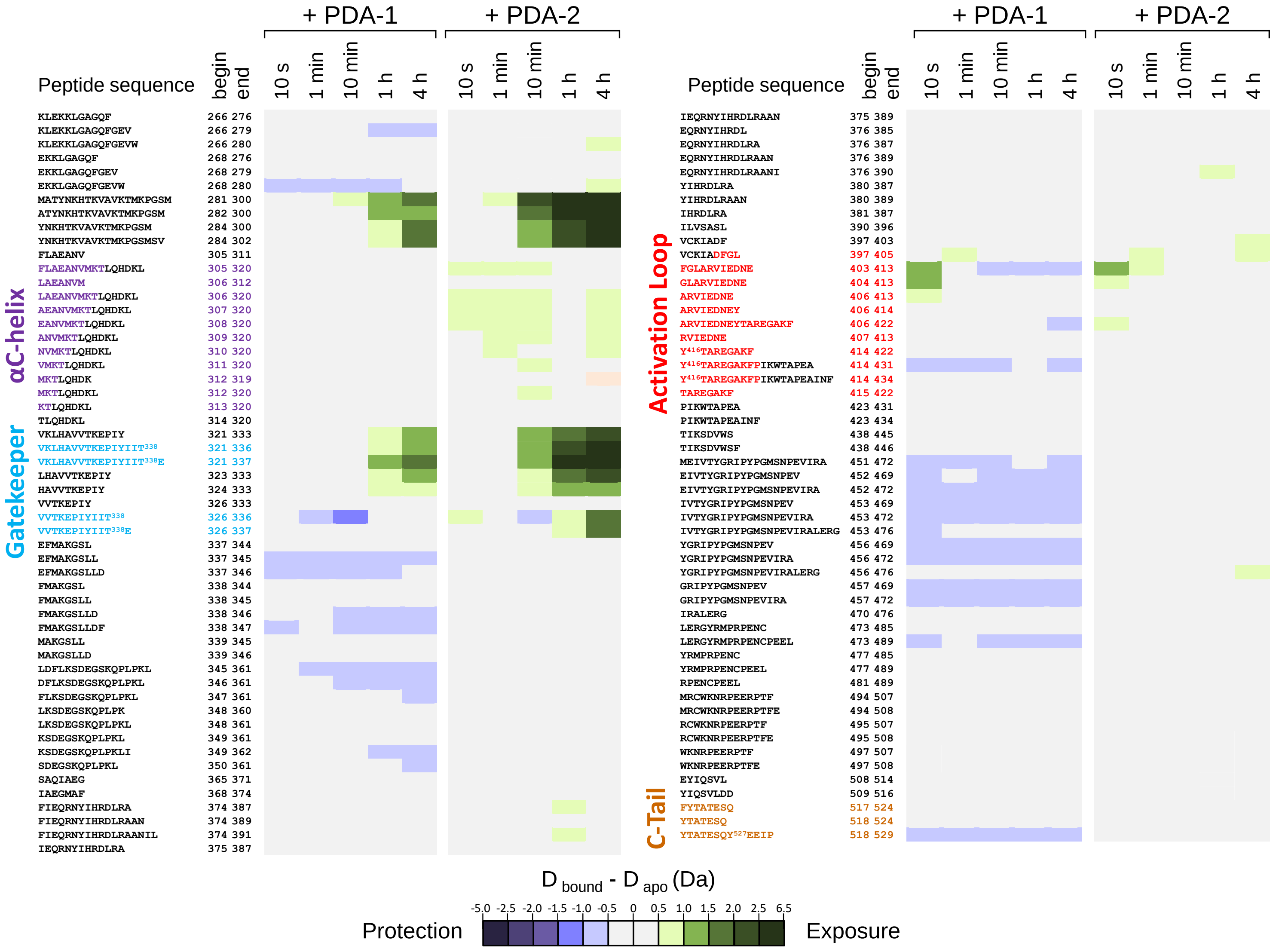

Figure S6B: HDX-MS difference maps for near-full-length Hck in the presence of PDA1 and PDA2. Data are shown for the kinase domain; SH3 and SH2 domains plus the SH2-kinase linker peptides are shown in Figure S6A. Peptides containing residues from the **αC-helix**, the **gatekeeper residue (Thr338)**, the **activation loop and autophosphorylation site (pTyr416)**, and the **C-terminal tail and negative regulatory phosphotyrosine (pTyr527)** are highlighted.

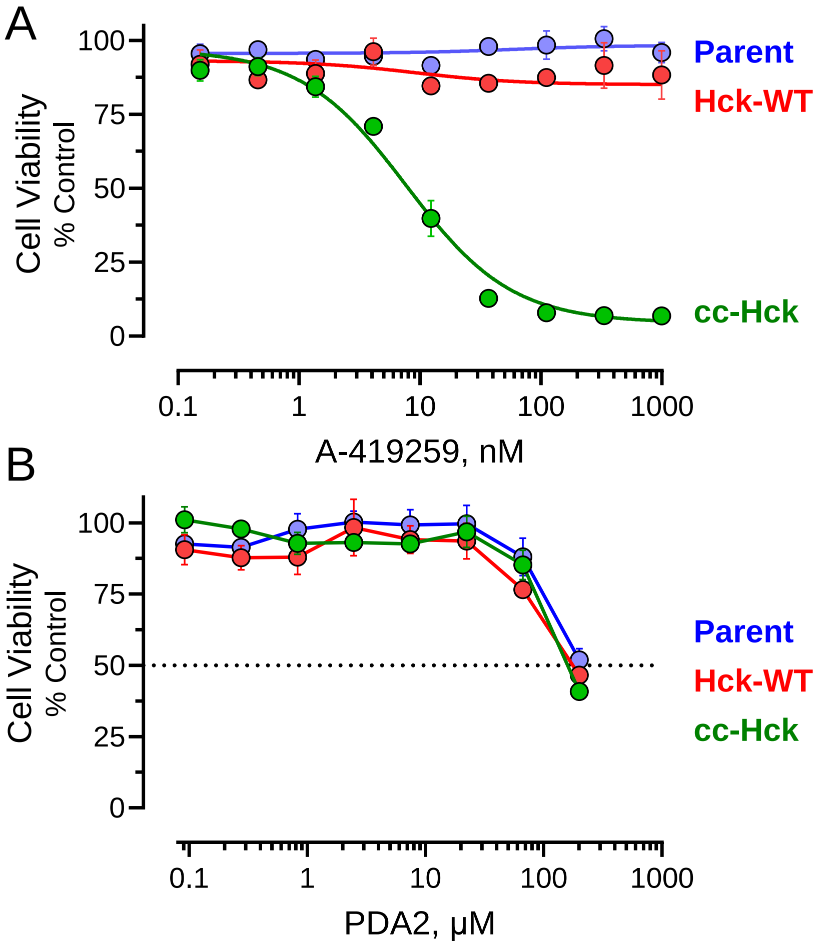

Figure S7: Effects of A-419259 and PDA2 on the viability of TF-1 cells expressing wild-type Hck and cc-Hck. Parental TF-1 cells or cell populations expressing wild-type Hck or an active cc-Hck fusion protein were incubated over a range of inhibitor concentrations or the DMSO carrier solvent (0.1%) alone as control. Parental cells and cells expressing wild-type Hck required GM-CSF for growth, while the TF-1/cc-Hck population acquired a cytokine-independent phenotype. Cell viability was determined 72 hours after addition of small molecules or vehicle using the CellTiter Blue cell viability assay (Promega). Results were normalized to the DMSO control values and are presented as mean percent control ± SE for triplicate determinations. A) Effects of orthosteric (ATP-site) inhibitor A-419259. B) Effects of PDA2, which is about 100-fold less potent than PDA1 (see main Figure 8) and shows no preference for any of the cell populations.

Literature cited in Supplement.

1. Kieken, F., Loth, K., van Nuland, N., Tompa, P., and Lenaerts, T. (2018). Chemical shift assignments of the partially deuterated Fyn SH2-SH3 domain. Biomol NMR Assign *12*, 117-122. 10.1007/s12104-017-9792-1.

2. Jung, J., Byeon, I.J., Ahn, J., and Gronenborn, A.M. (2011). Structure, dynamics, and Hck interaction of full-length HIV-1 Nef. Proteins *79*, 1609-1622. 10.1002/prot.22986 [doi].

3. Williamson, M.P. (2013). Using chemical shift perturbation to characterise ligand binding. Prog Nucl Magn Reson Spectrosc *73*, 1-16. 10.1016/j.pnmrs.2013.02.001.
